# *In vitro* inhibition of human leukemia THP-1 cells by *Origanum syriacum* L. and *Thymus vulgaris* L. extracts

**DOI:** 10.1186/1756-0500-7-612

**Published:** 2014-09-07

**Authors:** Basim M Ayesh, Abdalla A Abed, Doa’a M Faris

**Affiliations:** Medical Technology Department, Faculty of Science, Alaqsa University, Gaza, Palestine; Biology and Biotechnology Department, Faculty of Science, the Islamic University of Gaza, Gaza, Palestine P.O.Box 108,; European Gaza Hospital Laboratory, Ministry of Health, Gaza, Palestine

**Keywords:** Leukemia, THP-1 Cells, *Origanum syriacum*, *Thymus vulgaris*, Antiproliferative, Cytotoxic

## Abstract

**Background:**

Natural products including, traditional medicinal plants have emerged as a tempting alternative to conventional chemotherapeutic protocols of leukemia because of their minimum side effects and less documented drug resistance.

**Methods:**

Ethanol extracts were prepared from *Thymus vulgaris* L. and *Origanum syriacum* L. plants and investigated against the THP-1 leukemia cell line and freshly isolated peripheral blood mononuclear cells (PBMCs). The 3-[4,5-dimethylthiazol-2-yl]-2,5-diphenyl tetrazolium bromide (MTT) assay and the lactate dehydrogenase (LDH) assay were respectively used to determine the cellular viability and cytotoxicity in response to treatment with increasing extract concentrations.

**Results:**

Both extracts exhibited a concentration dependent reduction in viability of the THP-1 cells (IC_50_ = 2.126 mg/mL for *O. syriacum*, and 0.1569 mg/mL for *T. vulgaris*). *O. syriacum* was more potent against the PBMCs (IC_50_ = 0.4247 mg/mL), while *T. vulgaris* was moderately selective (IC_50_ = 0.3345 mg/mL with PBMCs and SI = 2.1). Only in *O. syriacum* the reduction in cells viability was caused by cytotoxic effect against leukemic cells (LC_50_ = of 9.646 mg/mL).

**Conclusion:**

*T. vulgaris* and *O. syriacum* are both antileukemic *in vitro. T. vulgaris* represents a potential selective cytostatic and safe target for future anticancer agents’ development. *O. syriacum* on the other hand is cytotoxic against the leukemia cell line THP-1.

## Background

Depending on its type, leukemia may be successfully treated with chemotherapy, radiation therapy, hormonal therapy, or bone marrow transplantation. However, chemotherapeutic agents are highly toxic to a wide range of normal body cells, and thus are associated with diverse side effects. In addition, multiple drug resistance is a major determinant of chemotherapy failure. Therefore, natural products including, traditional medicinal plants, have emerged as a tempting more tolerated alternative with minimum side effects.

The leaves of *Origanum syriacum* L. (Syrian Oregano), which belongs to the Lamiaceae family and inhabits a large area in the eastern Mediterranean, have been widely used in the traditional herbal medicine [1, 2]. Its composition and effects as antioxidant and antibacterial were extensively studied, but few were concerned with the antiproliferative and anticancer activities of this plant [3, 4].

*Thymus vulgaris* L. (thyme) is a member of the genus *Thymus* (Lamiaceae family) which is predominantly found in the Mediterranean region, Asia, Southern Europe and North Africa [5]. Extracts of *T. vulgaris* are traditionally used as anti-asthmatic, bronchodilator, antitussive, antispasmodic [6], antibacterial, and antifungal [7, 8]. In addition, these extracts have shown immunomodulating properties [9, 10]. Different studies proposed that extracts of *Thymus vulgaris* may have potential anticancer effects [11–15].

In this study, we investigated the antiproliferative and cytotoxic effects of extracts from *Origanum syriacum* and *Thymus vulgaris* on the acute monocytic leukemia cell line THP-1 and human peripheral blood mononuclear cells (PBMCs) isolated from normal controls.

## Materials

All cell culture reagents, media and supplements as well as cell viability kits and syringe filters were purchased from Sigma Aldrich (Israel). The THP-1 cell line was purchased from the European Collection of Cell Cultures (ECACC, UK). Cell culture flasks, well plates and other tissue culture plastics were purchased from Greiner Bio-One (Germany).

## Methods

### Collection of plant materials and preparation of extracts

*T. vulgaris* seeds were purchased from PlantiCo (Poland) and implanted for six months. *O. syriacum* were obtained from Al-Breem plantation (Khanyounus) and authenticated by Dr. Mohammad Abo-Oda (PhD plant taxonomy, Alaqsa University, Gaza). Aerial parts were collected, washed, air-dried under shade for 3 weeks, and then completely dried by oven at 40°C for 1 to 1.5 hour.

Both plant extracts were prepared by soaking the dried and grinded aerial parts in ethanol at room temperature with occasional mixing (7 days, 40% ethanol for *O. syriacum;* and 4 days, 90% ethanol for *T. vulgaris)* in a weight/solvent volume ratio of 1/10. Different ethanol concentrations were used according to the solubility preferences of the literature detailed effective compounds of each plant. The extracts were filtered under vacuum for three times, and dried by rotary evaporator at 40°C for *T. vulgaris* and 45°C for *O. syriacum*.

*O. syriacum* stock solution was finally prepared at a concentration of 200 mg of the dry extract/mL of RPMI-1640 medium with 10% fetal bovine serum (FBS). *T. vulgaris* stock solution was prepared at the same concentration in dimethyl sulfoxide (DMSO). DMSO was used because *T. vulgaris* dry extract contains high percentage of oils and does not dissolve in the aqueous RPMI-1640 medium. The stock solutions were stored at -20°C until used.

### Cell line culturing and maintenance

The THP-1 cell line was derived from the peripheral blood of a 1 year old male with acute monocytic leukemia. The cells were cultured in modified RPMI-1640 complete medium with 2.05 mM L-glutamine and 25 mM HEPES, supplemented with 10% heat inactivated FBS at 37°C in a humidified atmosphere with 5% CO_2_. Cell counts and viability estimation by trypan blue dye exclusion test were performed regularly. Throughout the study procedures, THP-1 cells were maintained in a logarithmic growth phase at a concentration between 10^5^-10^6^ cells/mL. Media feeding was performed periodically every 2–4 days.

### Isolation of human Peripheral Blood Mononuclear Cells (PBMCs)

PBMCs were isolated from sodium heparin anticoagulated venous blood of healthy donors using Sigma’s Histopaque-1077 as recommended. The donors’ consents were obtained before sample collection, and the procedure was approved by the local Helsinki committee of the Palestinian Health Research Council, according to the World Medical Association Declaration of Helsinki (approval reference No. 1-12/2012). The cells were suspended at a concentration of 10^6^ cells/mL in modified RPMI-1640 medium, with 2.05 mM L-glutamine and 25 mM HEPES, supplemented with 10% FBS, 5 μg/ mL phytohemagglutinin (PHA), 100 μg/mL streptomycin, and 100 U/mL penicillin.

### Cells preparation and extracts treatment

THP-1 cells in the exponential growth phase and viability of at least 95% and freshly isolated PBMCs with viability of at least 98% were used for the viability assays. The cells were seeded in 96-well plates at a density of 10^4^ THP-1 cells/well and 10^5^ PBMCs cells/well in 100 μL of the culture medium. Another 100 μL of the proper extract working concentration were added to the corresponding wells in triplicates, and the cells were incubated for 48 ± 1 hours in a humidified CO_2_ incubator at 37°C and 5% CO_2_.

The various extract working concentrations were prepared by dilution of the stock solutions in culture medium and filter-sterilization with 0.22 μm Millex-GP syringe filters. Corresponding DMSO concentrations were similarly prepared as vehicle controls for *T. vulgaris* extract.

### Cell viability assay

The THP-1 cells and PBMCs previously incubated with the proper extract or DMSO concentration were washed several times with 150 μL phosphate buffered saline (PBS) (pH = 7.2 - 7.6) and plate-centrifugation, to remove any residual extract color that may interfere with the colorimetric assay. The extract-free cells were finally suspended in 100 μL media with 10% heat inactivated FBS, and assayed for viability using the colorimetric MTT based *in vitro* toxicology assay kit following the manufacturer instructions. The assay measures the amount of the blue-colored formazan accumulated intracellularly following cleavage of the 3-(4,5-dimethylthiazol-2-yl)-2,5-diphenyltetrazolium bromide (MTT) reagent by mitochondrial dehydrogenases of the viable cells. The absorbance of the produced intracellular formazan which is proportional to the number of viable cells present was determined at 550 nm. Absorbance for background correction was determined at 620 nm. Absorbance of wells filled with media alone was used as a blank and untreated control wells were seeded with cells that incubated without any extracts. The percentage of cell survival was calculated after background absorbance correction and blank absorbance subtraction as follows: % Cell viability = 100 Χ Experimental well absorbance / untreated control well absorbance.

### Cytotoxicity assay

The THP-1 cells were treated with various extracts or DMSO concentrations. Cells in the positive control wells were treated with 1% Triton X-100 solution, and negative control wells cells were incubated in culture media alone. Blank wells contained the corresponding extract concentrations or Triton X-100 solution or media without cells. The lactate dehydrogenase (LDH) based *in vitro* toxicology assay kit was used to assay for cytotoxicity following the manufacturer instructions. The assay measures membrane integrity as a function of the amount of cytoplasmic LDH released into the medium. LDH reduces NAD into NADH, which is utilized in the reduction of a tetrazolium dye to colored formazan. The amount of formazan which is proportional to the amount of LDH release from dead cells was measured colorimetrically at 450 nm. Absorbance for background correction was determined at 620 nm.

The percentage of cell viability was calculated as follows: % Cell viability = 100 –% cell cytotoxicity. The % cell cytotoxicity = 100 X (experimental well absorbance –negative control well absorbance) / (positive control well absorbance –negative control well absorbance). All calculations were performed after background absorbance correction and blank absorbance subtraction.

### Statistical analysis

All experiments were performed in triplicates and each experiment was performed for two times. The results were expressed as a mean viability percentage ± standard deviation (SD). Dose response curves were prepared with Microsoft Office Excel 2007 software.

The data were analyzed using the GraphPad Prism software (Version. 6). The log (inhibitor) vs. response curve equation was used to determine the best fit and to determine the IC_50_ and LC_50_ values, the concentration that provokes a response half way between the maximal and the maximally inhibited cell viability. The obtained curves on leukemic and normal cells for each extract were compared with respect to their IC_50_/LC_50_ and slope and the P-value was determined with 95% confidence intervals. Two-regression curves comparison was performed by the F-test and a significant difference was obtained at P-values < 0.05.

## Results

### Effects of the extracts on THP-1 Cells and PBMCs viability

**Figure 1:**
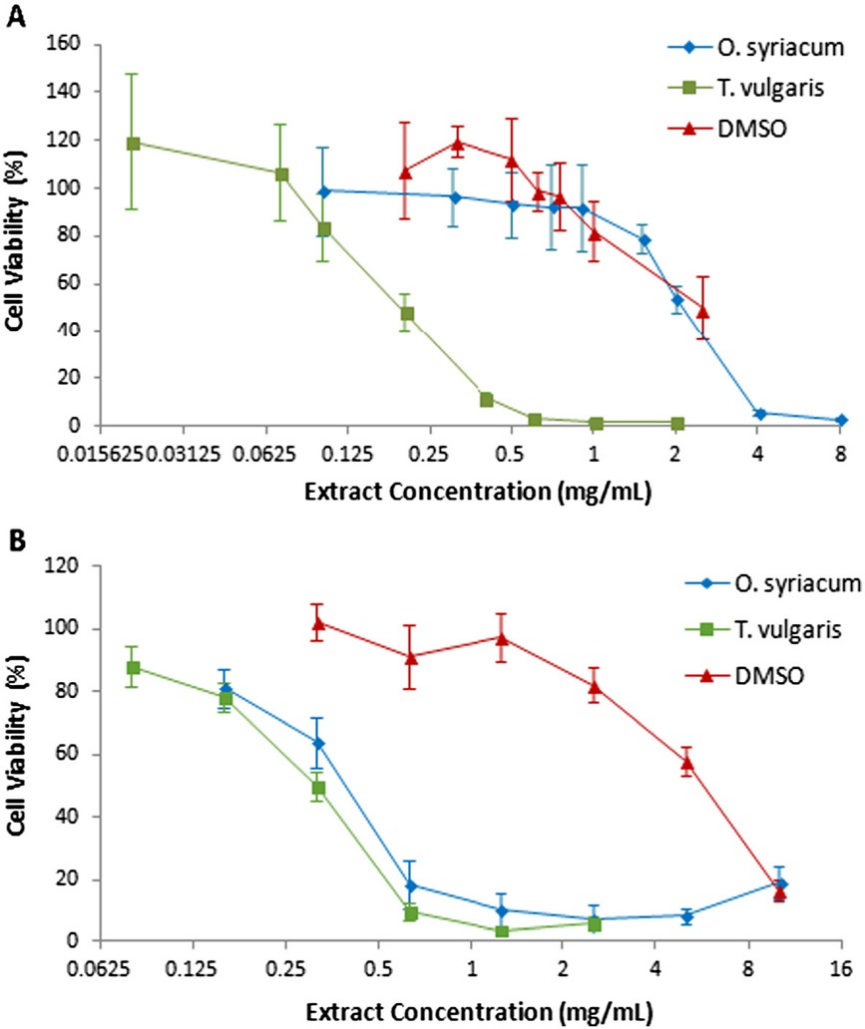
**Reduction of THP-1 cells and PBMCs viability in response to increasing extracts and DMSO concentrations.** Dose response curves of THP-1 cells **(A)** and PBMCs **(B)** were constructed after 48 h treatment with *O. syriacum* and *T. vulgaris* extracts and DMSO. The cells viability was assayed by the colorimetric MTT based assay. The X-axis is in logarithmic scale.

Both extracts *O. syriacum*, and *T. vulgaris* significantly reduced the number of viable THP-1 cells and PBMCs in a concentration dependent manner [Figure 1]. No effect was obtained with DMSO at the effective *T. vulgaris* extract concentrations. *T. vulgaris* extract effect was selective against leukemic cells with selectivity index (SI) of 2.1. An SI value <2 indicates a general nonspecific toxicity. On the other hand, the *O. syriacum* extract effect was more potent on normal cells than on leukemic cells. The IC_50_ values for THP-1 cells and PBMCs as well as selectivity indices are listed in Table 1.

**Table 1:** **IC**_**50**_ **values and selectivity indices (SI) of** ***O. syriacum*** **and** ***T. vulgaris*** **extracts**

Plant extract	IC _50_ (95% Confidence interval) in mg/mL		P-value	SI
	THP-1 Cells	PBMCs		
***O. syriacum***	2.126 (1.934 - 2.339)	0.425 (0.339 - 0.533)	< 0.0001	0.2
***T. vulgaris***	0.157 (0.133 - 0.186)	0.334 (0.311 - 0.359)	< 0.0001	2.1

All values were determined by GraphPad Prism nonlinear regression analysis. The Log (inhibitor) vs. response - Variable slope (four parameters) equation was used for best fit of all extracts curves. In all of these curves, the bottom was constrained to zero value. SI = IC_50_ of extract in the normal cell line/IC_50_ of the same extract in cancer cell line.

### Cytotoxic effects of the extracts on THP-1 cells

**Figure 2:**
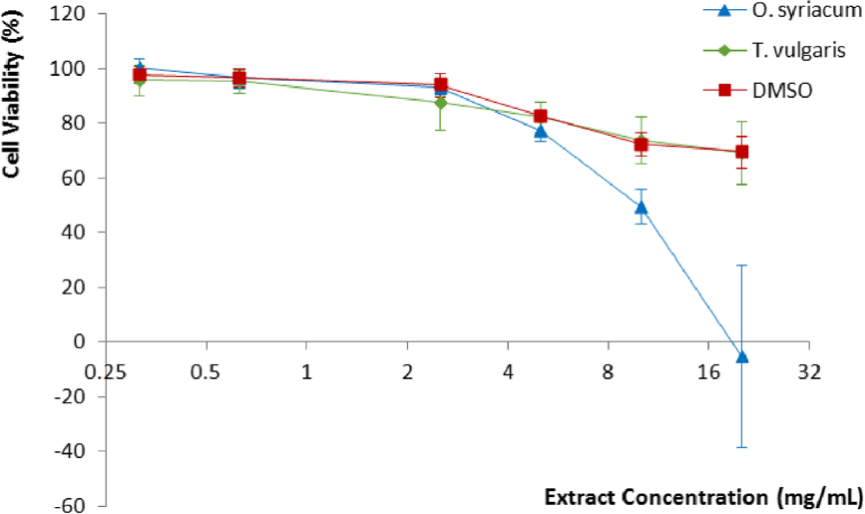
**Cytotoxic effect on THP-1 cells in response to increasing extracts and DMSO concentrations.** Dose response curves of THP-1 cells were constructed after 48 h treatment with *O. syriacum* and *T. vulgaris* extracts and DMSO. Cytotoxicity was determined by LDH based assay. The X-axis is in logarithmic scale.

The LDH assay was used in order to determine if the reduction in cell viability obtained with MTT assay was due to cytotoxicity or to an antiproliferative effect. *T. vulgaris* extract has not shown any cytotoxic effect against THP-1 leukemic cells compared to DMSO (P-value = 0.6520) [Figure 2]. *O. syriacum* extract on the other hand has shown significant cytotoxic effect against THP-1 cells with LC_50_ value of 9.646 mg/mL.

## Discussion

In this study, *T. vulgaris* exhibited a selective dose dependent inhibition of THP-1 proliferation (cytostatic activity) at the applied concentrations and exposure time rather than killing them (cytotoxic activity). These promising findings give hope that *T. vulgaris* may contain cancer therapeutic agents that, once purified, are more selective to leukemic cells than normal blood cells at relatively low concentrations. Furthermore, *T. vulgaris* is among the safest extracts used in herbal medicine, exhibiting only minor adverse effects [16].

Our results are consistent with other studies which demonstrated a dose dependent proliferation inhibition of human cancer cell lines from breast cancer, leukemia, cervical epithelial carcinoma, oral cavity squamous cell carcinoma, lung carcinoma and prostate carcinoma [11, 13, 14, 17]. *T. vulgaris* essential oil were previously shown to interfere with the transcription of genes involved in the cell cycle, cell death and cancer, namely, interferon signaling, N-glycan biosynthesis and extracellular signal-regulated kinase 5 (ERK5) signaling [13].

In this study, *O. syriacum* extract has resulted in both reduced viability and increased cytotoxicity of the THP-1 and PBMCs. This would have been a much more valuable finding if the effect of *O. syriacum* extract was selective against the leukemic cells.

To the best of our knowledge, no previous studies have assessed the effect of *O. syriacum* against leukemia, although few ones reported an antiproliferative effect of *O. syriacum* crude extract against cancer cell lines derived from breast adenocarcinoma and human cervical adenocarcinoma [3, 4].

The effects imposed by the plant extracts on the normal PBMCs may have been aided by the challenges they face in culture, particularly when the culture media composition is modified by many substances present in the crude extracts. Such challenges are expected to affect normal cells more than leukemic cancer cells, which have gained some growth advantages during their multistage progress toward cancer. In this regard, *T. vulgaris* was shown by others to be inhibitory on normal Vero cell line and PBMCs at high concentrations [12, 17]. Only one study was found in the literature to deal with *O. syriacum* cytotoxicity against the, supposed, normal diploid human embryonic fibroblasts MRC-5 cell line [18]. Like our results, the study showed that methanolic extracts of *O. syriacum* were cytotoxic against the MRC-5 cells with IC_50_ > 64 μg/mL. No other studies were found in the literature to investigate the cytotoxicity of the *O. syriacum* extracts against PBMCs.

*T. vulgaris* and *O. syriacum* essential oils cytotoxicity was investigated by others *in vivo* in Wistar rats, and no significant variations of hematological or biochemical parameters were noticed after the oral treatment with these essential oils compared to the control group treated with physiological solution [19].

## Conclusion

In conclusion, *T. vulgaris* and *O. syriacum* are both antileukemic *in vitro. T. vulgaris* represents a potential selective cytostatic and safe target for future anticancer agents’ development. *O. syriacum*, on the other hand, is cytotoxic against the leukemia cell line THP-1. Further *in vitro* and *in vivo* research has to be performed on the molecular mechanisms of their action as well as on the chemical identity of their active substances.
